# Positron emission tomography molecular imaging to monitor anti-tumor systemic response for immune checkpoint inhibitor therapy

**DOI:** 10.1007/s00259-022-06084-1

**Published:** 2023-01-09

**Authors:** Xiaoqing Xing, Qing Zhao, Jinyun Zhou, Rui Zhou, Yu Liu, Xiyi Qin, Mingrong Zhang, Yan Zhong, Jing Wang, Mei Tian, Hong Zhang

**Affiliations:** 1 Department of Nuclear Medicine and PET Center, The Second Affiliated Hospital of Zhejiang University School of Medicine, 88 Jiefang Road, Hangzhou, 310009 Zhejiang China; 2 Institute of Nuclear Medicine and Molecular Imaging of Zhejiang University, Hangzhou, China; 3 Key Laboratory of Medical Molecular Imaging of Zhejiang Province, Hangzhou, China; 4 Department of Advanced Nuclear Medicine Sciences, Institute of Quantum Medical Science, National Institutes for Quantum and Radiological Science and Technology, Chiba, 263-8555 Japan; 5 Human Phenome Institute, Fudan University, Pudong New Area, Shanghai, 201203 China; 6 College of Biomedical Engineering & Instrument Science, Zhejiang University, Hangzhou, China; 7 Key Laboratory for Biomedical Engineering of Ministry of Education, Zhejiang University, Hangzhou, China

**Keywords:** Positron emission tomography (PET), Immune checkpoint inhibitor, Immune response, Immune-related adverse events (irAEs)

## Abstract

Immune checkpoint inhibitors (ICIs) achieve a milestone in cancer treatment. Despite the great success of ICI, ICI therapy still faces a big challenge due to heterogeneity of tumor, and therapeutic response is complicated by possible immune-related adverse events (irAEs). Therefore, it is critical to assess the systemic immune response elicited by ICI therapy to guide subsequent treatment regimens. Positron emission tomography (PET) molecular imaging is an optimal approach in cancer diagnosis, treatment effect evaluation, follow-up, and prognosis prediction. PET imaging can monitor metabolic changes of immunocytes and specifically identify immuno-biomarkers to reflect systemic immune responses. Here, we briefly review the application of PET molecular imaging to date of systemic immune responses following ICI therapy and the associated rationale.

## Introduction

Immunotherapy is emerging as a significant progress in cancer treatment [1, 2]. As the most representative immunotherapy, immune checkpoint inhibitors (ICIs) have revolutionized the treatment landscape and have become first-line cancer therapies, especially anti-programmed death receptor-1 and its ligand (α-PD1/PDL1) and anti-cytotoxic T-lymphocyte antigen-4 (α-CTLA4) [3, 4]. Multiple ICI agents have been approved by the US Food and Drug Administration (FDA) for cancer treatment, including melanoma, non-small cell lung cancer (NSCLC), and renal cell carcinoma (RCC) [5, 6]. ICIs could overcome tumor-mediated T cell inhibition and activate T cell expansion by blocking immune checkpoint proteins [7, 8]. The effective anticancer immune response is supported by an increase in systemic immune activation, including peripheral and secondary lymphoid organs [8–10]. Nevertheless, a limited number of patients benefit from ICI, whereas many other patients still demonstrate disease progression, also bearing high costs and immune-related adverse events (irAEs) from ICI therapy [11, 12]. Therefore, exploring an effective detection strategy for systemic immune response caused by ICI agents is crucial for adjusting the therapeutic regimen, reducing costs, and alleviating side effects.

**Fig. 1:**
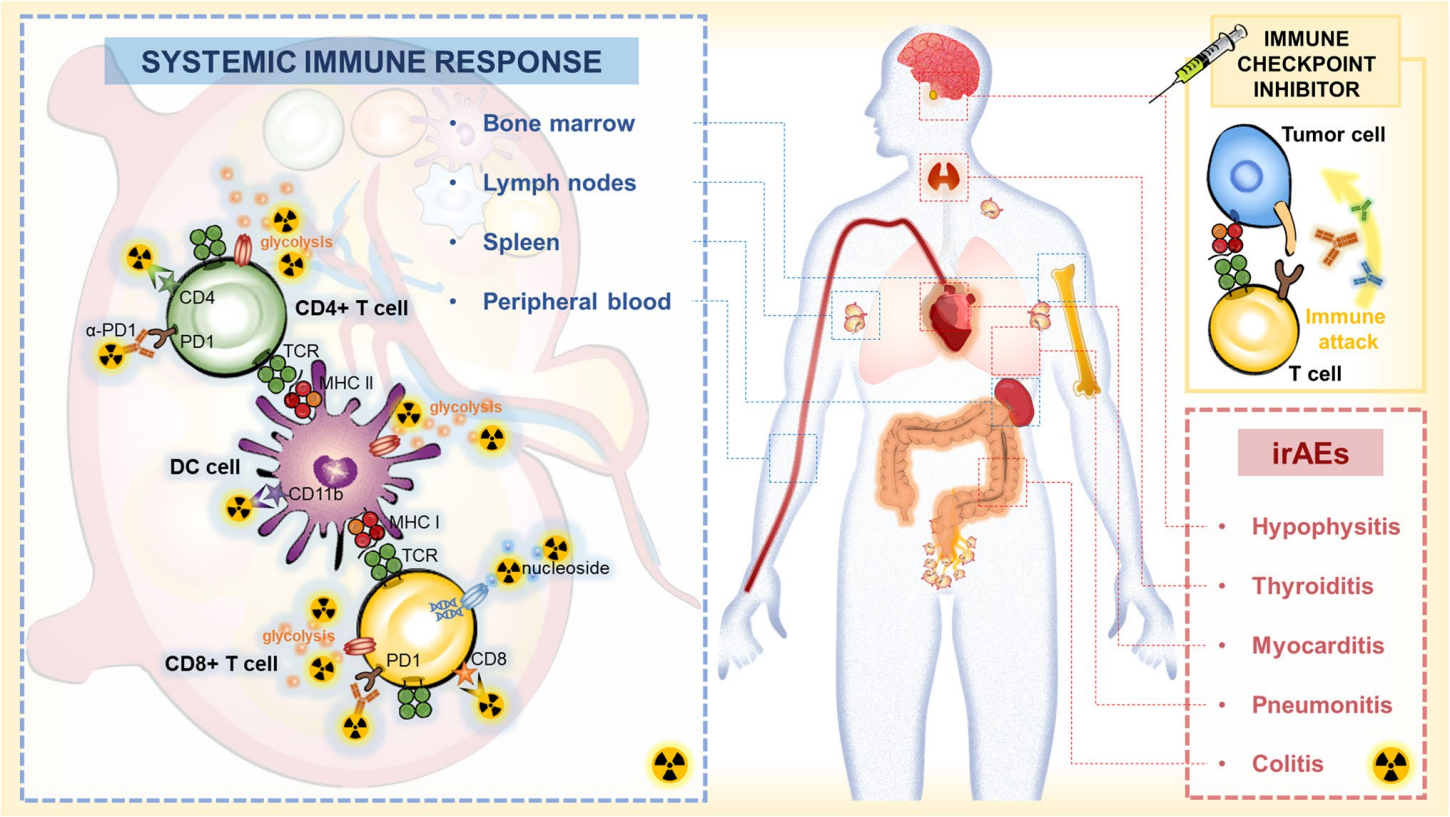
Systemic immune response to immune checkpoint inhibitors. When ICI treatment triggers effective immune activation, the complex changes including metabolic patterns and cellular dynamics occur in systemic immune cells. PET-based imaging can detect changes in metabolic markers or specific biomarkers of systemic immune responses to assess immune activation and predict irAEs after ICI therapy

To date, the assessment of biomarkers by a histopathological specimen from surgery is performed in clinical practice. However, the invasiveness of the procedure and the localized specimens limits its application [13]. Positron emission tomography (PET) can provide a noninvasive macroscale (>1 mm) evaluation of the biological processes with spatial and temporal quantitative analysis as a generalized evaluation system in transpathology [14, 15]. Moreover, the advantages of different imaging examinations on the anatomy, metabolism, and cellular pathology are merged into the hybrid imaging techniques involving PET/computed tomography (PET/CT) and PET/magnetic resonance imaging (PET/MRI) [15]. Thus, PET-based imaging is regarded as a potential method for evaluating systemic immune responses caused by ICI agents [8, 9]. This review briefly illustrates the current status of PET-based imaging in the monitoring of systemic immune response on ICI therapy and discusses the future opportunities for the new methods of immunotherapy response assessment (Fig. 1).

## Immune checkpoint inhibitors

Unprecedented progress has been made in cancer immunotherapy over the past decade. Currently, blocking antibodies against immunosuppressive receptors is the most widely used immunotherapy approach [10]. In the cancer-immunity cycle model, immune checkpoints serve as inhibitory regulators, helping tumor cells to evade immune surveillance [16, 17]. The rationale for targeting immune checkpoints is to block immune checkpoints to stimulate the immune system’s endogenous anticancer response [18]. The host’s immune system resists tumor cells, which is a multistep immune cell–malignant cell interaction. Host antigen-presenting cells (APCs) bind to T cell receptors (TCRs) on T cells through major histocompatibility complex-I (MHC-I) on APCs to activate naive host T cells against tumor antigens [19]. Numerous monoclonal antibodies against immune checkpoints have been widely used in clinical practice, especially α-CTLA4, α-PD1, and α-PDL1.

CTLA4 is expressed on regulatory T cells (Tregs) and transiently on conventional T cells upon activation. CTLA4 not only recognizes the endogenous antigenic peptide-major histocompatibility complexes, and then kills the target cells, but is also able to suppress T cell priming and activation via competitively blockading CD28-CD80/CD86 binding [20–22]. Ipilimumab and tremelimumab are two human monoclonal α-CTLA4 antibodies applied for the treatment of melanoma, NSCLC, RCC, lymphoma, head and neck squamous cell carcinoma, hepatoma, etc. [23]. Although there are some limitations in the clinical use of α-CTLA4 therapy, some clinical studies have found that the combined regimen of α-CTLA4 and other treatment modalities (e.g., radiotherapy, chemotherapy) can effectively enhance the α-CTLA4 antitumor effect. The clinical success of α-CTLA4 combination treatment has also attracted interest for the development and exploration of other ICIs [10].

PD1 is expressed on T lymphocytes and other immune cells. As one of the ligands of PD1, the binding of PDL1 to PD1 leads to an inhibitory signal on the activation of T cells. Therefore, preventing the binding of PD1 to PDL1 contributes to T cell activation and immune response enhancement [24–26]. There are currently a variety of human monoclonal antibodies that antagonize the PD1/PDL1 inhibitory axis, such as nivolumab, cemiplimab, and pembrolizumab, which bind to PD1, and atezolizumab, avelumab, and durvalumab, which block the interaction with PDL1. Nivolumab and pembrolizumab are currently approved for the treatment of various types of solid and liquid tumors. α-PD1 treatment is approved as a tissue diagnostic modality for any metastatic or unresectable tumor that exhibits mismatch repair deficiency or microsatellite instability due to its excellent therapeutic efficacy [27]. Additionally, the novel combination strategies of α-PDL1 with other treatment methods are also undergoing clinical trials to seek more effective anticancer treatment regimens.

## Systemic immune response to immune checkpoint inhibitors

To provide systemic protection, the tumor-immune interactions occur at systemic sites, such as peripheral blood, bone marrow (BM), lymph nodes (LNs), and spleen, in addition to local immune responses in the tumor microenvironment (TME). A series of complex interactions between the tumor and the global immune system leads to impaired tumor recognition and elimination by cytotoxic T lymphocytes (CTL). Therefore, the systemic immune responses are essential for successful cancer ICI immunotherapy.

Peripheral blood collection from patients with cancer has been widely used in clinical practice to screen for tumor antigen-specific T cells. In addition, there are immunosuppressive immune cells in peripheral blood [28–30], such as Tregs, which secrete immunosuppressive cytokines (IL-10 and TGF-β) and express inhibitory checkpoint molecules (CTLA4), thus exerting an inhibitory effect on the proliferation of activated CD4+ CD25− and CD8+ T cells in the circulation [28]. During immunotherapy, T cell clonal exchange between tumor and blood is vital. The cancer-related immune response can extend to lymphoid organs, with extensive expansion of lymphocytes.

As the primary lymphoid organ, the BM maintains the host immune system and provides progenitor cells for all leukocytes [29]. Notably, tumor antigen-specific T cells tend to circulate and accumulate in BM compared to peripheral blood [30]. The presence of CD11c+ dendritic cells (DCs) within the BM captures, processes, and presents antigens to naive CD4+ and CD8+ T cells. This process generates the primary immune response [31, 32].

The spleen is a key organ for local and systemic immune regulation, serving as a secondary lymphoid organ for recycling red blood cells, capturing antigens, filtering blood, and activating innate and adaptive immune responses against antigens [33, 34]. Furthermore, the spleen provides a suitable site for the expansion of myeloid cells and the recruitment of tumor-associated macrophages and granulocytes to the primary tumor. Circulating DCs within the spleen acquire the antigens from blood or tissue, and perform antigen presentation [35]. After antigen recognition, CD4+ and CD8+ T cells are redistributed into non-lymphoid tissues. Subsequently, monocytes enter the tumor matrix and produce tumor-associated macrophages [36]. In addition, the immature myeloid cells and DCs of the spleen upregulate the expression of the PDL1 and downregulate the expression of CTLA4 [37].

LNs of tumor patients have higher levels of DCs and CD8+ T cells, and the interaction between DCs and T cells appears to be very active in LNs [38, 39]. In contrast, tumor-draining LNs contain immunosuppressive immune subsets (e.g., checkpoint molecules in T cells, Tregs, and myeloid-derived suppressor cells (MDSCs)) that suppress T cell responses by interfering with tumor-specific sensitization of T cells in tumor-draining LNs of tumor-bearing hosts [40, 41]. LNs provide a pre-metastatic location where malignant cells can remain dormant until recurrence or metastasis. Therefore, tumor-draining lymph nodes are a major site of enhanced early T cell activation after ICI therapy.

T cell activity through ICI therapy represents an important breakthrough in treating human cancer. ICI treatment alters the TME, the systemic immune microenvironment, and the immune cell composition, thereby resulting in tumor inhibition [10, 42]. Unfortunately, only a small fraction of patients treated with ICI are responsive [43, 44]. Therefore, further research is required to monitor systemic immune responses occurring in circulation and lymphoid organs, which can reflect or predict the efficacy of ICI treatment.

## PET molecular imaging assessment of systemic immune response caused by ICI therapy

At present, the most common biomarkers for predicting the efficacy of ICI therapy are PDL1 and PD1 expression measured by immunohistochemistry (IHC). However, the IHC assay is challenged by multiple limitations including sampling errors and tumor heterogeneity. PET molecular imaging may potentially circumvent these issues by using specific radiotracers and allow for non-invasive, whole-body, dynamically monitored tumor and immune cell characteristics, providing a framework capable of assessing immune activation. Increasing tracer uptake indicates efficient immune remodeling in metabolic PET imaging. Molecular imaging targeting PD-1/PD-L1 with radiolabeled antibody can predict the patient response to PD-1/PD-L1 blockade therapy in immune PET imaging. Selecting the optimum treatment strategy may overcome ICI resistance.

## Metabolic PET imaging

### ^18^F-FDG

**Fig. 2:**
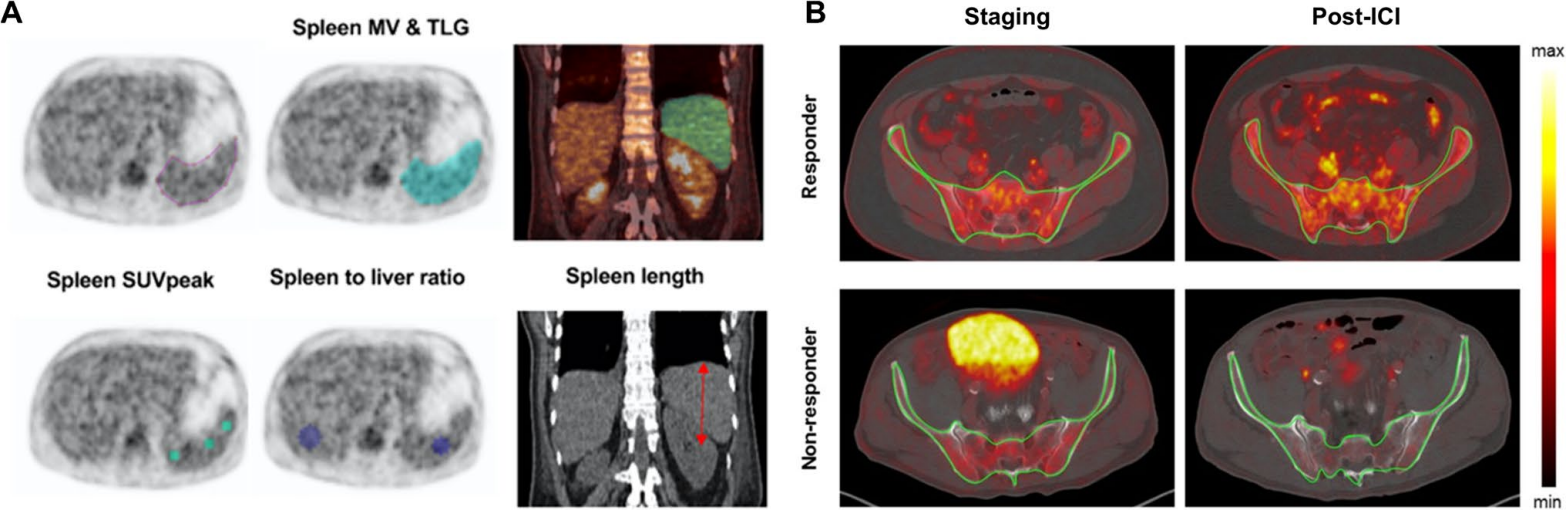
^18^F-FDG PET imaging can be used to monitor metabolic patterns in lymphoid organs for the evaluation of systemic immune response after ICI therapy. **A** Methodology for measurement of uptake in the spleen to assess ICI outcome. **B** Representative PET/CT images showing the baseline scan and the follow-up scan of BM after the initiation of ICI treatment. The significantly enhanced ^18^F-FDG uptake within the bone was a consequence of successful treatment. (The researches were originally published in Eur J Nucl Med Mol Imaging. 2021 Jul;48(8):2573-2585 [52] and Theranostics. 2020 Jan 1;10(2):925-937 [53], respectively)

PET with the radiotracer [18F]2-deoxy-2-fluoro-d-glucose (^18^F-FDG) is routinely used in clinical practice to find primary tumors and metastases. Additionally, ^18^F-FDG PET provides information on glucose metabolism to assess immune activation [45, 46]. ICI therapeutic strategies have contributed to altering the outcome and prognosis of melanoma patients via activating the immune system [47]. Therefore, the potential of ^18^F-FDG to evaluate the systemic immune response against melanoma caused by ICI treatment should be considered [48]. A recent study shows that the systemic immune response of ICI-treated patients could be reflected by ^18^F-FDG PET 2 weeks after treatment initiation in lymphoid organs. The ^18^F-FDG uptake of the spleen of responders and non-responders was significantly different, in which the effective systemic immune response in patients can be detected as an increased spleen activity. Moreover, changes in bone marrow ^18^F-FDG uptake showed the same trend as spleen volume but were less pronounced [49]. In order to predict the prognosis of melanoma patients receiving ICI treatment by parameters efficiently reflecting the immune activation by ^18^F-FDG PET images, Prigent et al. reviewed the PET parameters of 29 patients at 1, 3, and 6 months after ICI treatment (Fig. 2A). The authors reported that the spleen to liver ratio of ^18^F-FDG uptake (SLR) could be considered an outcome predictor in melanoma patients undergoing ICI therapy. Compared with baseline, an increase in SLR_mean_ greater than 25% was related to poor treatment response [50]. ^18^F-FDG PET also could illustrate an immunologic reconstitution by evaluation of splenic glucose consumption in α-PD1-treated Hodgkin lymphoma patients. Increased spleen metabolism in ICI responders was observed in a preliminary study by Dercle et al. Notably, FDG uptake by healthy spleen tissue was more pronounced in responders, indicating an efficient immune remodeling [51].

In addition, a dual-center retrospective study also aimed to identify melanoma patients who could benefit from α-PD1 therapy with ^18^F-FDG PET scanning. The bone marrow to liver SUV_max_ ratio of glucose metabolism (BLR), which is associated with hematopoietic cells and systemic immunosuppression, also could be used to predict progression-free survival (PFS) and overall survival (OS) of melanoma patients treated with α-PD1 [54]. Furthermore, the glucose metabolism of bone marrow was significantly increased in patients with clinical response to ICI agents compared to patients with resistance to ICI (Fig. 2B). This effect was observed in the axial skeleton before treatment initiation and on follow-up examinations, whereas only a slight trend was observed in the appendicular skeleton on follow-up ^18^F-FDG PET scans. This difference may be due to extensive activation of the BM [53]. In NSCLC patients, a lower BLR was found to reflect a satisfactory prognosis [55], BM is the main lymphoid organ, and its hematopoietic function is necessary for effective anti-cancer immunotherapy. For adults, BM is not only an important hematopoiesis organ for innate and adaptive immune cells but also an important storage site of immune cells, including bone marrow cells (e.g., MDSC), plasma cells, naive T cells, memory T cells, and regulatory T cells [56]. Increased hematopoietic requirements accelerate the bone marrow metabolism, leading to increased glucose uptake as detected by ^18^F-FDG PET [57, 58]. In a multicenter retrospective study, Seban et al. found that baseline ^18^F-FDG PET/CT parameters such as SLR and BLR helped identify patients who were resistant to ICI treatment. BLR was associated with most blood-based inflammatory indices, including platelet-to-lymphocyte ratio (PLR), C-reactive protein (CRP), and systemic immune-inflammatory index (SII). In contrast, SLR was only associated with CRP [59]. There is currently insufficient evidence linking immunotherapy effects to pathophysiological and immunological mechanisms of the spleen and BM. Further research is needed to better understand the relationship between splenic, BM glucose metabolism, and pro-inflammatory and immunosuppressive environments, which will also facilitate the application and development of PET-based imaging in immunotherapy. Although the ^18^F-FDG PET technique is widely used and can reflect ICI efficacy, the different ^18^F-FDG PET parameters recommended from different studies have not reached a consensus on assessing the systemic immune response to ICI therapy [48–50, 53–55, 60, 61]. Thus, these innovative and promising assessment methods should be validated in a large and multi-center population.

### New metabolic-PET imaging probes

Since glucose metabolism in cells traced by FDG is a non-immune cell-specific process and FDG activity in the bone marrow and spleen often changes whatever the treatment given or even without treatment, other markers that more accurately characterize the complexity of the immune environment are needed (Table 1), such as 2′-deoxy-2′-^18^F-fluoro-9-β-d-arabinofuranosylguanine (^18^F-FAraG), 2′-deoxy-2′-[18F]fluoro-β-d-arabinofuranosylcytosine (^18^F-FAC), 2-chloro-2′-deoxy-2′-^18^F-fluoro-9-β-d-arabinofuranosyl-adenine (^18^F-CFA), and [18F]-fluoro-3′-deoxy-3′-l-fluorothymidine (^18^F-FLT).

**Table 1:** Metabolic PET imaging for ICI-relative systemic immune response against cancer

Application	Comments	Radiotracer	Main finding	Author (year)
glucose Consumption	The most commonly used PET imaging method is to monitor changes in cellular glycolysis in clinic. The increase in glycolysis that accompanies activation of the immune system can be reflected by ^18^F-FDG PET imaging, but this approach lacks cell specificity.	^18^F-FDG	SLR_mean_ measured by ^18^F-FDG PET is recommended as an indication in ICI-treated melanoma patients	Prigent et al. [52] (2021)
			Effective prognostic biomarker information could be provided by BLR combined with high MTV, concerning tumor metabolic characteristics and reflecting the immune system in advanced NSCLC patients under ICI treatment.	Lang et al*.* [55] (2021)
			The ^18^F-FDG PET/CT imaging monitoring the glucose metabolism in the lymphatic organs might be regarded as a preclinical and clinical tool to differentiate responders and non-responders.	Schwenck et al. [53] (2020)
			An effective systemic immune response can be observed in the splenic ^18^F-FDG PET in ICI-treated patients, notably early stage of treatment.	Seith et al. [62] (2020)
			BLR parameter could provide an early prognosticator of ICI treatment outcome by ^18^F-FDG PET scans.	Seban et al*.* [50] (2019)
DNA synthesis	With the potential to specifically respond to T lymphocytes, PET imaging in lymphoid organs is more advantageous. However, the spatial resolution and cellular sensitivity of PET imaging need to be improved.	^18^F-F-AraG	^18^F-F-AraG has the potential to become an efficacy approach for assessment of ICI outcome timely.	Levi et al. [63] (2019)
		^18^F-FAC	This combination of advanced MRI and PET imaging could provide an effective method to monitor glioblastomas patients treated with immunotherapy and to distinguish disease progression from pseudo-progression.	Antonios et al. [64] (2017)
		^18^F-CFA		
		^18^F-FLT	^18^F-FLT PET could be used for mapping cell proliferation in secondary lymphoid organs after anti-CTLA4 treatment in patients with melanoma.	Ribas et al. [65] (2010)

*SLR*, spleen-to-liver ratios; *NSCLC*, non-small cell lung cancer; *MTV*, metabolic tumor volume; *BLR*, bone marrow-to-liver ratios

^18^F-FAraG, a T cell–specific PET reagent, can be used as an imaging biomarker to measure the response to ICI therapy. Studies have demonstrated that ^18^F-FAraG accumulates in activated human CD8+ T cells, and the ^18^F-FAraG uptake in immunotherapy responders was significantly higher than those prior to immunotherapy. However, it was not statistically significant in non-responders at the same time point. Furthermore, the signal in tumor-draining lymph nodes played a key role in assessing α-PD1 treatment response [63]. In addition, ^18^F-FAraG PET reflects tumor CD8+ profiles prior to initiation of any therapy and tracks TME immunomodulation during chemotherapy-induced immune priming [66]. Overall, ^18^F-FAraG demonstrates potential as a systemic immune clinical monitoring tool to evaluate the body’s immune response and predict the effect of immunotherapy [67].

Antonios et al. reported two PET markers, ^18^F-FAC and ^18^F-CFA, focusing on deoxycytidine kinase substrates in DNA synthesis to reflect the systemic immune activation. However, ^18^F-FAC is not suitable for clinical application due to the rapid decomposition of cytidine deaminase in humans. Fortunately, ^18^F-CFA is more appropriate for human imaging and, when applied in combination with high spatial resolution MRI imaging, can be used to observe the infiltration of specific immune cells [64, 68, 69].

Molecular imaging using the PET probe ^18^F-FLT can visualize cellular proliferation in secondary lymphoid organs following CTLA4 blockade in metastatic melanoma patients, with the effect being pronounced in the spleen. However, since the diameter of lymph nodes is usually less than 1 cm, the sensitivity and resolution of PET imaging are insufficient, so its application in tumor-draining lymph node (TDLN) remains to be explored. The study demonstrated that tremelimumab at 15 mg/kg every 3 months released the CTLA4 cell cycle checkpoint in most patients, as evidenced by increased ^18^F-FLT uptake in the spleen after administration [65]. Although these probes are not absolutely specific to immune cells, changes in their accumulation throughout the body may indicate diseases and provide early biomarkers. The direct comparison of ^18^F-FAC with ^18^F-FDG and ^18^F-FLT showed that ^18^F-FAC had better selectivity for lymphoid organs such as the thymus, spleen, and lymph nodes [70]. It is necessary to directly compare ^18^F-FAC with ^18^F-FDG, ^18^F-FAraG, and ^18^F-FLT in the detection of immune response in various cancers in the future.

### Immuno-PET imaging

As an emerging molecular imaging modality, immuno-PET not only possesses the inherent high sensitivity of PET imaging, but also possesses excellent target specificity. Immuno-PET has become the optimal method to specifically image tumor markers, immune checkpoints, immune cells, etc. Table 2 summarizes the immuno-PET radiotracers that can be used to assess systemic immune responses.

**Table 2:** Immuno-PET imaging for ICI-relative systemic immune response against cancer

Radiotracer	Tumor	ICI Treatment	Main finding	Author (year)
^68^Ga-NOTA-Nb109	Melanoma	α-PDL1	The tracer has the potential for assessing PDL1 status in tumors and evaluating the ICI therapeutic effect via PET imaging.	Lv et al. [71] (2020)
^89^Zr-nivolumab	Lung cancer	Nivolumab	The tracer could allow for in vivo PET imaging, through targeting of localized activated T cells expressing PD1.	England et al. [72] (2018)
^89^Zr-nivolumab	NSCLC	Nivolumab	89Zr-nivolumab and 18F-BMS-986192 PET/CT could be potentially used in noninvasively assessing PD(L)1 expression in NSCLC patents.	Niemeijer et al. [73] (2018)
^18^F-BMS-986192	NSCLC	Nivolumab		
^89^Zr-atezolizumab	Bladder cancer, NSCLC, TNBC	Atezolizumab	The tracer uptake could be an effective predictor of response to atezolizumab treatment against tumors by evaluating PDL1 expression in the tumor and multiple lymphoid tissues.	Bensch et al. [74] (2018)
^89^Zr-pembrolizumab	Melanoma, NSCLC	Pembrolizumab	^89^Zr-pembrolizumab tumor uptake was higher in patients with response to pembrolizumab therapy. ^89^Zr-pembrolizumab also reflected uptake in lymphoid tissues and at sites of inflammation.	England et al. [75] (2017) Kok et al. [76] (2021) Niemeijer et al. [77] (2022) Van der Veen et al. [78] (2020)
^64^Cu-NOTA-PD(L)1	Melanoma	α-PDL1 + radiation therapy	Whole-body high-resolution and high-sensitivity PET imaging using the new radiotracers can reveal PD(L)1 expression in spleen and lymph nodes.	Hettich et al. [79] (2016)
^64^Cu-DOTA-PD1	Melanoma	α-PD1	This radiotracer could be used to evaluate the prognostic value of PD1 and could aid in predicting response to ICI treatment.	Natarajan et al. [80] (2015)
^124^I-JS001	Melanoma, Urologic cancer	JS001	^124^I-JS001 was a safe tracer for PET with acceptable dosimetry, and the PET/CT results showed a favorable biodistribution. PET/MR could detect liver lesions more sensitively than PET/CT.	Huang et al. [81] (2020) Wang et al. [82] (2021)
^89^Zr-DFO-CD4 ^89^Zr-DFO-CD8a	TNBC, colon cancer, kidney cancer, Melanoma	Sym021	These radiotracers could be used to predict response to Sym021 treatment via assessment of CD4+and CD8a+ status.	Kristensen et al. [83] (2019)
^89^Zr-VHH	Colon cancer	α-PD1	^89^Zr-VHH immuno-PET could be used to monitor theα-PD1 treatment responses and provide potential targets for intervention to enhance the anticancer immune response.	Rashidian et al. [84] (2019)
^64^Cu-169cDb	Mammary adenocarcinoma	α-PD1	^64^Cu-169cDb PET could effectively detect the distribution of CD8*+* T cells in normal organs and tumors resulting from ICI immunotherapy.	Seo et al. [85] (2018)
^89^Zr-DFO-CD3	Colon cancer	α-CTLA4	A CD3 PET radiotracer was developed to evaluate the ability of PET imaging to predict immune response to α-CTLA4 therapy.	Benjamin et al. [86] (2016)

*NSCLC*, non-small cell lung cancer; *TNBC*, triple-negative breast cancer

In recent years, PET-based imaging approaches using radiolabeled anti-PD1/PDL1 antibodies have shown detection advantages over pathological examinations [87]. First, PET imaging is non-invasive and can assess receptor expression in all metastases throughout the body; second, it can also be used to non-invasively assess the expression of various markers of chronic inflammation. Third, the detection results are quantitative and reproducible, and are suitable for evaluating the dynamic changes of PD1/PDL1 expression [88]. Therefore, PET imaging with therapeutic antibody-based PD1/PDL1 checkpoint tracers can provide valuable information to determine the efficacy of ICI treatment and systemic immune response [71, 73, 75–80].

Given that nivolumab showed great binding affinity to PD1 expressed on the surface of stimulated T cells, ^89^Zr-Df-nivolumab was developed to evaluate the imaging of PD1-expressing T cell infiltrates in a humanized mouse model of lung cancer. In this study, a human peripheral blood lymphocyte-severe combined immunodeficiency (hu-PBL-SCID) model was created using immunodeficient NOD/SCID/IL2γc null (NSG) mice. Specific accumulation of the tracer was observed in stimulated PD1-expressing T cells both in vitro and in vivo, indicating activation of T cells and increased expression of PD1 in PBL mice. Notably, the ^89^Zr-Df-nivolumab imaging agent primarily targets and localizes to the tumor (non-direct imaging) rather than the actual tumor cells [72]. Natarajan et al. developed a mouse PET anti-PD1 tracer ^64^Cu-DOTA-PD1 for tumor-infiltrating lymphocytes in a melanoma transgenic mouse model. Lymphocytes were imaged to specifically display PD1 expression in tumors and the spleen; besides, they will seek to correlate PD1 expression levels to the PET signal in the future [80]. The ^68^Ga-labeled nanobody tracer ^68^Ga-NOTA-Nb109 has successfully achieved specific and noninvasive imaging of PDL1 expression in a mouse model of melanoma, which can accumulate in tumors of high PDL1 expression. Analysis of the expression of immune checkpoints in patients before ICI treatment can effectively avoid ineffective treatment and improve the success rate of ICI treatment [71]. A novel radiotracer, ^64^Cu-NOTA-PD(L)1, was developed by Hettich et al. for high-resolution and high-sensitivity PET imaging of PD1 and PDL1 in immunocompetent mice. In this study, PD1 and PDL1 were noninvasively detected mainly in secondary lymphoid organs (spleen and lymph nodes) in naive mice. This may reflect the function of the PD1/PDL1 checkpoint in inducing peripheral T cell self-tolerance and limiting the induction of effector T cells [79]. The upregulation of extralymphatic tissue under inflammatory conditions suggests that it induces T cell depletion, limiting the activity, survival, and expansion of effector T cells, and protecting peripheral tissues from immune-mediated damages [81, 82]. Interestingly, this study also detected PDL1 in mouse brown adipose tissue (BAT), confirming its immunological relation to BAT [79].

**Fig. 3:**
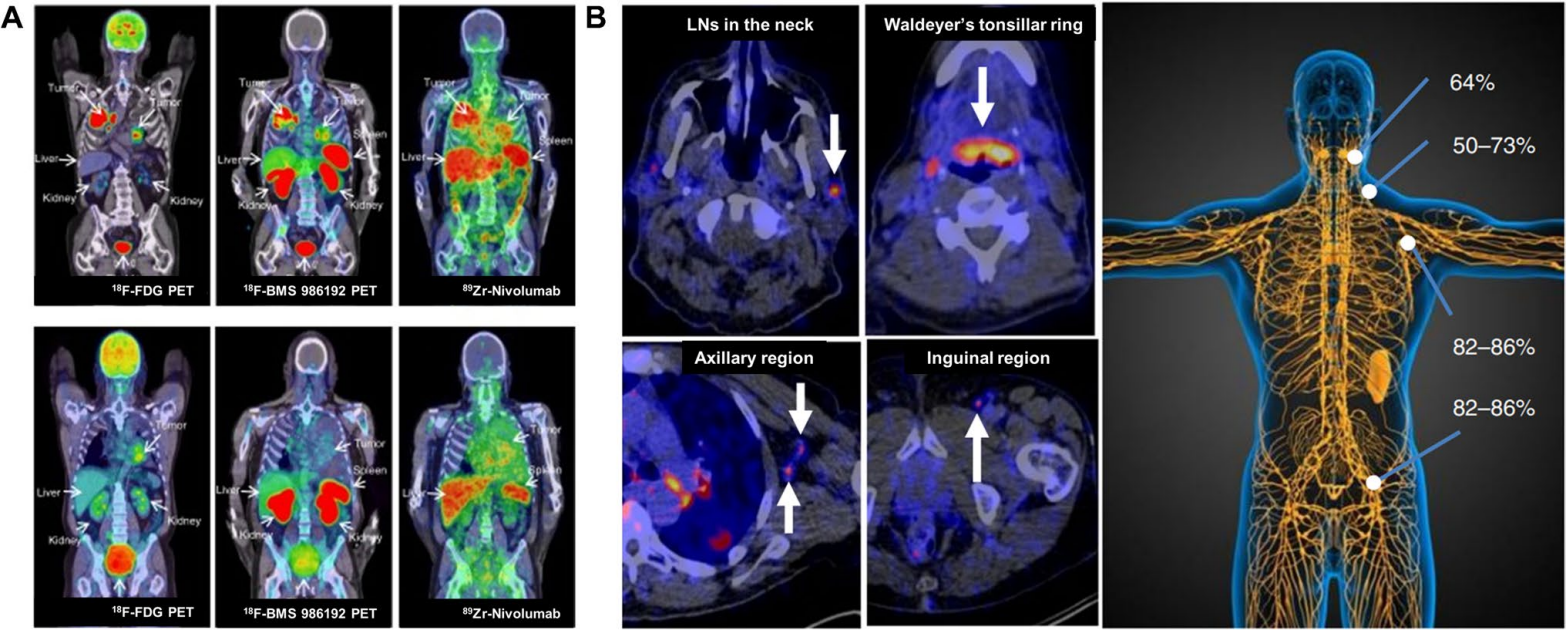
Whole-body PD(L)1 PET-imaging for the assessment of ICI therapy response. **A** Representative images of two patients detected by ^18^F-FDG PET, ^18^F-BMS-986192 PET, and ^89^Zr-Nivolumab PET to demonstrate heterogeneous tracer uptake. **B** Percentage of patients with ^89^Zr-atezolizumab uptake in healthy lymphoid tissue (right) and examples of ^89^Zr-atezolizumab uptake in the different regions (left). (The researches were originally published in Nat Commun. 2018 Nov 7;9(1):4664 [73] and Nat Med. 2018 Dec;24(12):1852-1858 [74])

Recently, therapeutic antibody-based PD1/PDL1 checkpoint tracers have also been applied in clinical practice. In preclinical studies, PET imaging and biodistribution studies demonstrated high uptake of ^89^Zr-pembrolizumab in tissues containing human immune cells, including the spleen, lymph nodes, and bone marrow. The systemic biodistribution of ^89^Zr-pembrolizumab showed high PD1-mediated uptake in lymphoid tissues such as the spleen, lymph nodes, and bone marrow, as well as moderate tumor uptake [75, 78]. The first-in-human study of ^89^Zr-pembrolizumab PET/CT in patients with advanced-stage NSCLC included patients who received two ^89^Zr-pembrolizumab injections and four PET/CT scans. The study found that ^89^Zr-pembrolizumab had a good safety profile, with only one adverse event possibly related to immunity. ^89^Zr-pembrolizumab was more absorbed in responders compared to non-responders, but did not correlate with PDL1 or PD1 immunohistochemistry [77]. In another clinical study to predict whether patients would benefit from α-PD1 therapy, 18 patients with advanced or metastatic melanoma and NSCLC were injected with ^89^Zr-pembrolizumab for PET scanning up to 3 times on days 2, 4, and 7. The study concluded that the optimal dose was 5 mg of pembrolizumab, and the optimal time point for a PET scan was day 7 [79]. Uptake of ^89^Zr-pembrolizumab was highest in the spleen. Moreover, uptake of ^89^Zr-pembrolizumab was also found in Waldeyer’s circle, normal LNs, and sites of inflammation, reflecting the presence of PD1 in normal tissues and sites of inflammation. The uptake of ^89^Zr-pembrolizumab in tumor lesions correlated with tumor response, PFS, and OS. In summary, the major sites of T cell distribution can be observed by ^89^Zr-pembrolizumab PET imaging [77]. The ^18^F-BMS-986192 and ^89^Zr-Nivolumab probes were also developed, and for the first time in humans, whole-body imaging was performed in patients with advanced NSCLC prior to treatment with nivolumab. They have the potential to be effective imaging biomarkers for the non-invasive assessment of PDL1 and PD1 expression, respectively, with accurate quantitative assessment. Crucially, no severe adverse events associated with these two novel probes were found during tracer injection and PET scanning (Fig. 3A). The good biosafety of the probes has laid a solid foundation for their clinical use, but larger samples are needed to confirm the current results [73]. In addition, a novel clinical-grade PET tracer, ^124^I-JS001, was recently reported to assess human PD1 expression in PET imaging in S180 allografts of humanized PD1 C57BL/6 mice. JS001 (toripalimab) is a humanized IgG monoclonal antibody that strongly inhibits PD1 [81]. Subsequently, the research team carried out a pilot clinical translational research. The study enrolled patients with pathologically confirmed melanoma or urinary system cancer who underwent ^124^I-JS001 PET/CT and PET/MRI. The ^124^I-JS001 probe demonstrated good biosafety, and no adverse events occurred during the experiment. The spleen and liver of the selected patients showed high uptake, and both primary tumors and metastases showed a certain uptake of ^124^I-JS001. These results suggest that the ^124^I-JS001 probe combined with PET imaging may be an effective tool for clinical screening of PD1-positive patients for tumor immunotherapy [82]. Furthermore, the first-in-human pilot study of ^89^Zr-atezolizumab probe application (NCT02453984 and NCT02478099) was conducted in a total of 25 patients with malignancies, including locally advanced or metastatic bladder cancer, NSCLC, or triple-negative breast cancer (TNBC), 22 of whom completed 4 PET scans and received atezolizumab treatment until disease progression (PD). ^89^Zr-atezolizumab imaging signal effectively reflects PDL1 expression at sites of inflammation and in various normal lymphoid tissues (Fig. 3B), and tracer uptake appears to be a strong predictor of response to atezolizumab treatment, indicating PFS and OS. However, the results should still be confirmed with a large patient population study and then combined with other clinical data to optimize the treatment response prediction model [74].

**Fig. 4:**
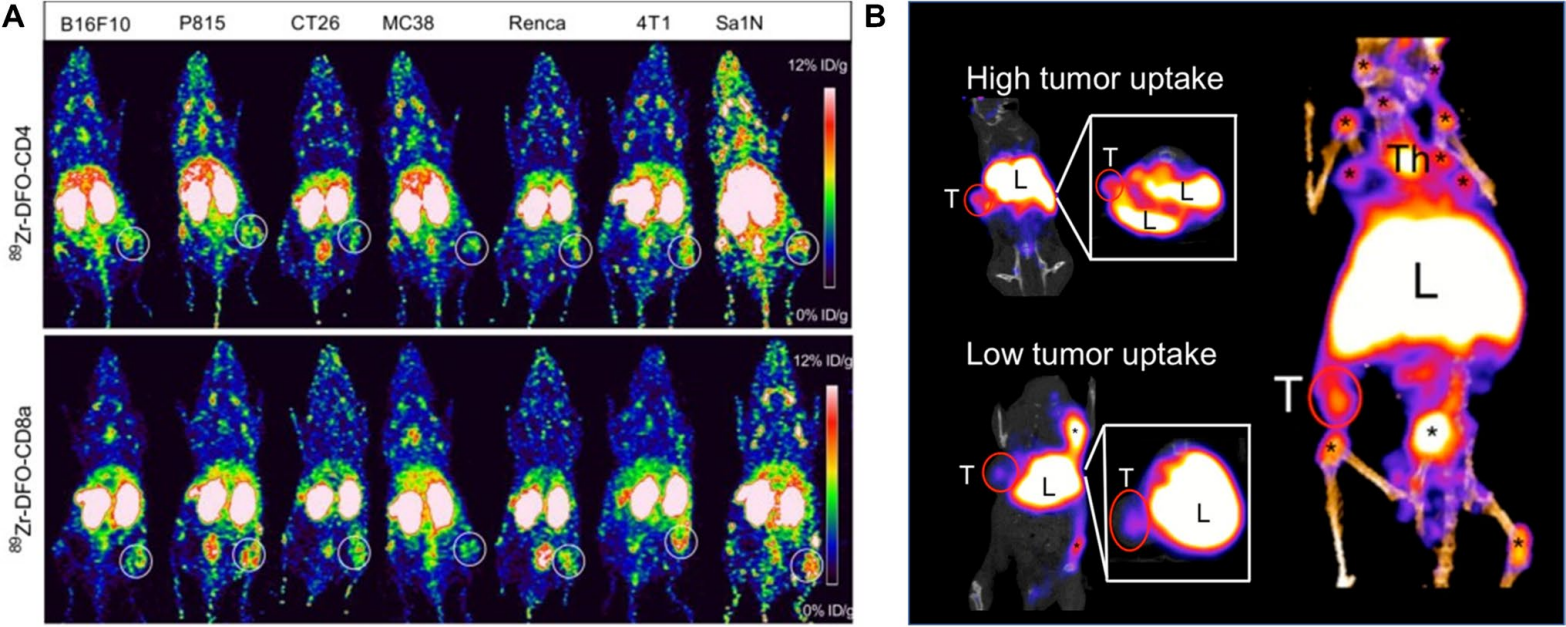
New methods to target immunotherapy-related biomarkers. **A** Representative coronal maximum intensity projection PET images of ^89^Zr-DFO-CD4 and ^89^Zr-DFO-CD8a for different cancer models. **B** Representative coronal ^89^Zr-DFO-CD3 PET images of α-CTLA4-treated tumor-bearing mice. Uptake could be observed in different tissues. *, LNs; L, liver; T, tumor; Th, thymus. (The researches were originally published in Theranostics. 2019 Oct 18;9(26):8221-8238 [83] and J Nucl Med. 2016 Oct;57(10):1607-1611 [86])

Imaging of PD1/PDL1 can be used to trace T cells activated by ICI treatment, visualizing the pathological processes and interplay between T cell activation, homing, and tumor residency. However, the use of immune checkpoint antibodies as imaging agents also has limitations. Immune checkpoint pathway receptors are expressed heterogeneously in multiple regions of the body, a biological property that complicates imaging strategies. Therefore, it is necessary to develop new methods to target immunotherapy-related biomarkers. Immunogenicity absence of a tumor is a major cause for the failure of ICI therapy, limiting the infiltration of tumor-infiltrating lymphocytes (TILs). PET-specific tracers for the non-invasive detection of CD4+ and CD8a+ cells were developed and preclinically studied. Seven mouse tumor models (4T1, CT26, B16F10, P815, MC38, Renca, Sa1N) were established for ^89^Zr-DFO-CD4 and ^89^Zr-DFO-CD8a PET/CT imaging to assess the effect of Sym021 (a humanized PD1 antibody cross-reactive with mouse PD1) treatment (Fig. 4A). ^89^Zr-DFO-CD4 was found to act as a predictor of tumor growth response and overall survival for Sym021. These two probes are effective for the systemic evaluation of CD4+ and CD8a+ lymphocyte status and the prediction of response to ICI therapy, and can potentially be investigated in clinical research [83]. In vivo study using ^89^Zr-labeled PEGylated single-domain antibody fragments (nanobodies or VHHs) for PET imaging has also been performed to assess intratumoral CD8+ T cells and CD11b+ myeloid cells after α-PD1 treatment. This imaging agent can effectively assess the response of ICI treatment by the distribution of CD8+ and CD11b+ cells in the tumor [84]. Although the study provides a novel method for the assessment of ICI treatment response, the research focuses on the tumor immune microenvironment, and the evaluation of systemic immune activation by specific PET imaging still requires exploration. ^64^Cu-169cDb PET imaging has also been developed to assess the distribution of CD8+ T cells in tumors and normal organs. Changes in tumor-infiltrating CD8+ T cells induced by ICI immunotherapy can be accurately visualized and quantified by ^64^Cu-169cDb PET imaging. Furthermore, the authors found that treatment-related hypertrophy of the liver and spleen reduced the circulation time of the imaging probe, providing an indication of off-target effects associated with immunotherapy protocols [85]. Benjamin et al. reported the evaluation of the ^89^Zr-DFO-CD3 probe for CTLA4 immunotherapy in the mouse colon cancer xenograft model (Fig. 4B). Since CD3 is part of the T cell receptor complex and a global marker of T lymphocytes, CD3 represents a more abundant target compared to a subpopulation marker. Thus, CD3 PET imaging agents targeting T cells may be a stronger predictor. High uptake of the PET tracer in α-CTLA4-treated tumor-bearing mice is a predictive biomarker of the immune response [86].

## PET molecular imaging for assessing immune-related adverse events

Immune-related adverse events (irAEs) are defined as collateral, inflammatory side effects caused by immune system activation after ICI treatment [89]. They usually occur when checkpoint inhibitors cause an immune-based immune attack on normal tissues in the first weeks to months after therapy but also at any time, even after treatment has stopped [90], subsequently inducing autoimmune and autoinflammatory responses in many organs. Various organ systems could be affected by irAEs, particularly the gastrointestinal tract and endocrine glands, and cutaneous, less common but potentially more serious cardiovascular, pulmonary, and hematologic systems are involved [91, 92]. Although the precise mechanism of irAEs remains unclear and may vary depending on the type of ICI therapy and the organ affected, there is emerging evidence that T cell activation is a hallmark of irAEs, particularly colitis [93]. In addition, cross-reactivity between anti-tumor T cells and similar antigens on healthy cells seems to account for some irAEs, such as vitiligo [94], myocarditis [95], and hepatitis [96]. Recently, augmented T cell–B cell interactions can lead to the production of autoantibodies, and the abnormal interactions have been associated with autoimmunity, resulting in thyroiditis [97], myasthenia gravis [98], and type 1 diabetes mellitus [99]. Given that ICIs are monoclonal antibodies against molecules expressed by both immune cells and other tissues, some irAEs may also result from complement-mediated direct injury from ICIs [100]. Additionally, considering the sudden onset and fatal toxicity of irAEs, it is critical to recognize and treat irAEs as early as possible.

Though irAEs are usually diagnosed clinically, some irAEs seem like asymptomatic and only diagnosed by imaging modalities. Recent studies have shown the potential of PET-based imaging to detect irAEs before clinical diagnosis [9]. On FDG PET/CT, irAEs are characterized by increased uptake of FDG in the affected organ, and subsequent reduction in uptake indicates remission of acute inflammation, meaning that it can be radiographically manifested to a certain extent [101]. Therefore, ^18^F-FDG PET/CT is a sensitive method for recognizing inflammatory processes that can be reflective of irAEs (Table 3) [102].

**Table 3:** PET molecular imaging for assessing immune-related adverse events

Radiotracer	Tumor	ICI	% irAEs after ICI therapy	Main finding	Author(year)
^18^F-FDG	Melanoma Lung cancer	Anti-PD-1	83%	^18^F-FDG detected 83% of irAEs in patients treated with anti-PD1 and medical imaging can direct targeted treatment.	Ahmed et al. [103] (2018)
^18^F-FDG	Metastatic melanoma	Anti–CTLA-4	NA	Radiological findings of immune-related adverse events were associated with significant clinical outcomes of anti-CTLA-4 therapy.	Yulia et al. [104] (2011)
^18^F-FDG	Metastatic melanoma	Vemurafenib Ipilimumab	43.75%	The presence of irAEs signs in ^18^F-FDG PET, such as colitis and arthritis, may be related to the benefits of immunotherapy.	Christos et al. [60] (2018)
^18^F-FDG	Bladder cancer	Pembrolizumab	39%	Increased uptake of ^18^F-FDG was detected in 39% of the patients after immunotherapy consistent with inflammatory changes.	Laura et al. [105] (2020)
^18^F-FDG	Malignant melanoma; malignant lymphoma; renal cell carcinoma	Nivolumab Pembrolizumab Ipilimumab	NA	Early development of thyroiditis detected by ^18^FDG-PET/CT may specifically represent an indicator of early response to immunotherapy.	Tomomi et al. [106] (2018)
^18^F-FDG	Advanced melanoma	Ipilimumab Nivolumab	66%	^18^FDG-PET/CT is an approach for long-term outcomes assessment in advanced melanoma treated with first-line ICIs.	Amir et al. [107] (2020)
^18^F-FDG	Metastatic melanoma	Anti-PD-1 or anti-CTLA-4	31%	Increased uptake of ^18^F-FDG can predict the development of irAEs in metastatic melanoma patients after ICI therapy and represents a potential quantitative imaging biomarker for irAEs.	Nežka et al. [102] (2022)
^18^F-FDG	Cancers	Atezolizumab and avelumab	21%	Increased diffuse uptake of ^18^F-FDG in thyroid may predict the onset of thyroiditis, which is a biomarker of anti-tumor immune response.	Anupam et al. [108] (2020)
^18^F-FDG	Lung cancer	NIVOLUMAB	33%	^18^F-FDG PET/CT provides predictive information for the development of thyroiditis with subsequent hypothyroidism.	Naghmehossadat et al. [109] (2018)
^18^F-FDG	Malignant melanoma Lung cancer Other cancers	NIVOLUMAB	17.2% 14% 20.8%	Increased uptake of ^18^F-FDG in thyroid related to good prognosis in lung cancer but might uncertain in malignant melanoma.	Ichiro et al. [110] (2019)
^68^Ga-NOTA-GZP	Foxp3-DTR-GFP mice bearing MC38 tumors	Anti-CD137 or anti-PD1 + anti-CTLA-4	54–76%	The higher uptake of ^68^Ga-NOTA-GZP in organs affected by irAEs is correlated with the presence of GZB and immune infiltrates confirmed by histology.	Carolina et al. [111] (2021)
^68^Ga-DOTATOC	ICI-related myocarditis	ICI	NA	The use of ^68^Ga-DOTATOC PET/CT combined with immune correlator is a highly sensitive method to detect ICI-associated myocarditis, especially in the early stages of myocardial inflammation.	Sarah et al. [112] (2021)

Mekki et al. conducted a medical imaging analysis of irAEs in fifty-three malignant patients treated with anti-PD1 and found that the detection rate of irAEs by ^18^F-FDG PET/CT was as high as 83% [103]. Evidence suggests that irAEs may reflect the burst immune activity required for antitumor effects, and irAEs are associated with higher response rates to immunotherapy [60, 104, 105]. A prospective study investigated the relationship between response to pembrolizumab and irAEs induced by ^18^F-FDG PET/CT in patients with early-stage bladder cancer. Notably, patients with irAEs not only had a higher complete pathological response rate and lower stage rate than patients without irAEs, but also had a higher baseline composite positive score and tumor mutational burden. Although these patients also showed longer PFS and better prognosis, the findings were not statistically significant due to the small sample size [105]. In addition, 43.75% of patients with metastatic melanoma on immunotherapy showed radiographic signs of irAEs by ^18^F-FDG PET/CT, and the PFS of these patients was significantly longer than that of patients without irAEs (*p* = 0.036). This finding suggests that the PET/CT appearance of irAEs may be associated with more effective immunotherapy [60]. Therefore, the relationship between FDG accumulation in tumors, lymphocyte-rich organs, irAE-related organs, and the development of irAEs was investigated. The irAEs detectable by ^18^F-FDG PET imaging indicated a favorable immunotherapy outcome. Especially in thyroiditis patients, early detection of irAEs was associated with effective ICI therapy [106]. However, larger cohorts are needed to validate this finding.

Moreover, the specific role of PET imaging in monitoring irAEs has been investigated. In a retrospective study evaluating the ^18^F-FDG PET findings of irAEs in patients with advanced melanoma on first-line nivolumab plus ipilimumab treatment, 80% of irAEs were identified by post-treatment ^18^F-FDG PET/CT examination and clinically confirmed, while 7% of irAEs were detected by ^18^F-FDG PET/CT before the appearance of clinical symptoms [107]. Thus, PET can be applied to monitor the occurrence of multisystem irAEs and may be used for the early detection of irAEs. By retrospectively analyzing 58 patients with malignant melanoma treated with α-PD1 or α-CTLA4, Hribernik et al. established a quantitative bioassay of ^18^F-FDG PET/CT for the diagnosis of irAEs using a convolutional neural network (CNN) approach. The authors analyzed the correlation of SUV percentiles (SUVX%), a novel quantitative imaging biomarker of ^18^F-FDG uptake in target organs, with clinical irAE status. The optimal percentiles for identifying irAEs were as follows: intestinal (SUV: 95%), lungs (SUV: 95%), and thyroid (SUV: 75%). This study provides more accurate PET-based prediction information for the occurrence of irAEs in melanoma patients treated with ICIs [102]. The introduction of artificial intelligence (AI) analysis methods provides quantitative and high-throughput information for PET imaging to effectively detect irAEs.

ICI treatment has become a major cause of endocrinopathies. An increased incidence of thyroid dysfunction (7–21%) has been found with α-PD1 therapy [97, 108, 113]. Thereby, several studies have focused on the analysis of thyroid-related irAEs with PET-based imaging. A retrospective study by Eshghi et al. investigated whether FDG uptake parameters could predict thyroiditis and subsequent hypothyroidism in lung cancer patients receiving nivolumab immunotherapy. The study found diffusely increased thyroid FDG uptake (including SUV_mean_, SUV_max_, and total lesion glycolysis) is predictive of thyroid dysfunction [109]. Thyroid irAEs in cancer patients treated with PDL1 inhibitors were characterized as new-onset hypothyroidism, thyrotoxicosis, and worsening of pre-existing hypothyroidism in a study conducted at the Mayo Clinic in Rochester, Minnesota. Diffuse increases in thyroid FDG uptake occurred in 71% of thyroid irAEs by analyzing PET images from 91 patients [108], consistent with the findings of Eshghi et al. [109]. Similar results were obtained by reviewing cancer patients treated with nivolumab at the Kyoto University Hospital [110].

Recently, a granzyme B–targeted novel PET imaging agent, ^68^Ga-NOTA-GZP, was developed by Ferreira et al. to identify malignant irAEs. GZP-PET imaging was performed in Foxp3 DTR GFP mice bearing MC38 tumors. Multiple organs, including the colon, spleen, and kidney, were successfully imaged in a model of irAEs, confirming the potential role of targeted granzyme B imaging in irAEs [111]. A more recent study reported that ^18^F-FDG PET/CT failed to reflect the intratumoral immune response, whereas ^68^Ga-NOTA-GZP PET/CT showed convincingly the real-time status of intratumoral immune response throughout the immune therapy, highlighting the future potential of GZP PET for irAE assessment [114].

^68^Ga-DOTA (0)-Phe (1)-Tyr (3)-octreotide (^68^Ga-DOTATOC) PET/CT has been shown to be valuable in the early diagnosis of ICI-related myocarditis, and has potential as a highly sensitive method. Another advantage of ^68^Ga-DOTATOC PET/CT is the absence of physiological uptake in normal myocardium and does not require an extensive carbohydrate-free diet, and consequently overcomes the main limitation of ^18^F-FDG in myocarditis, making it suitable for emergency setting [115]. Surprisingly, pathological uptake of ^68^Ga-DOTATOC was also found in the skeletal muscle of 83% of patients with myositis, suggesting an additional advantage of this method in assessing the full spectrum of myositis [112]. Although ^68^Ga-DOTATOC PET/CT has achieved good results in the detection of concomitant myositis, larger cohort studies are needed for validation. A growing number of studies have focused on the detection of irAEs by PET-based imaging. Such imaging could be used as a tool for early detection and monitoring of irAEs. However, the validity mechanism of irAE prediction still needs to be explored, and the standardization of inspection remains to be established to develop additional specific probes that reduce the chance of progression to life-threatening irAEs by enabling early intervention, and guide treatment development and administration.

## Conclusion and perspectives

Although ICI treatments play an important role in TME, their activation of systemic immune responses cannot be ignored. The immune activation of systemic lymphoid organs triggered by blocking checkpoints can be reflected by PET imaging through changes in metabolites and specific biomarkers, which is termed as “transpathology.” Therefore, PET-based imaging provides a new perspective for comprehensively evaluating the efficacy, prognosis, and side effects of ICI therapy.

However, the development of PET molecular imaging is much slower than tumor immune therapy; only few systemic immune responses are visualized compared to the in-depth molecular mechanism of immune therapy. Thus, the application of PET assessment in systemic immune response still has greater potential to be tapped. To gain a deeper understanding of specific immune cell and biomarker changes induced by ICI treatment, more specific probes need to be developed to provide clinicians with more detailed diagnostic information. The reliability of some candidate PET imaging parameters still needs to be verified by prospective, large samples, and even multicenter studies. Furthermore, PET-based AI has been proven to be feasible and effective for the assessment of immunotherapy in small patient samples. The collaborative efforts between multi-disciplinary oncology teams have the great potential to overcome the challenges associated with the use of these techniques and better stimulate clinical translation. With further progress of PET-based imaging, guiding cancer immunotherapy and systematic immune response into the era of precision medicine would hold promising prospects.

## Data Availability

Not applicable
